# A Rare Case of Survival from Inferior Vena Cava Injury

**DOI:** 10.7759/cureus.6907

**Published:** 2020-02-07

**Authors:** Ahmad Usman, Ayesha Shabbir, Abdul Basit

**Affiliations:** 1 Department of General Surgery, Nishtar Medical University and Hospital, Multan, PAK

**Keywords:** inferior vena cava, firearm, injury, venorrhaphy, trauma, resuscitation

## Abstract

Inferior vena cava (IVC) is the most commonly injured abdominal vessel in blunt and penetrating abdominal traumas, and its injury carries a very high rate of mortality. Hemodynamic instability at presentation, poor response to resuscitation, the anatomical level of venacaval injury, low Glasgow Coma Score, and concomitant vascular and visceral injuries are the main factors predicting the outcome of the patient. The primary surgical intervention needed is the control of hemorrhage followed by the repair of IVC defect, which may be done by venorrhaphy, ligation, use of patch or grafts, and other complicated procedures. Each of these techniques carries its own merits and demerits. This case report is of a patient who survived an infrarenal tear of IVC caused by a firearm injury that was repaired by venorrhaphy at a hospital of Pakistan with limited cardiac and endovascular facilities.

## Introduction

The inferior vena cava (IVC), being retroperitoneal in position and safeguarded by multiple abdominal viscera, has pretty less likelihood of being ruptured in cases of trauma, with the reported incidence of 0.5%-5% in penetrating and 0.6%-1% in blunt abdominal trauma [1]. However, among the abdominal vessels, it is the most commonly injured vessel in trauma, accounting for 25% of the abdominal vascular injuries [2]. In the case of firearm injuries of the abdomen, one in 50 bullets strikes the IVC [3]. Despite all the advancements in perioperative services, mortality remains exceptionally high, reported up to 65% in IVC injuries [4]. That is why a case of IVC injury always poses a taxing problem for the trauma surgeon, especially in hospitals with limited facilities like most hospitals in Pakistan. This case is of a patient who was successfully treated after an IVC tear in a limited resource hospital of Pakistan.

## Case presentation

A 28-year-old patient presented to the Accident and Emergency Department of Nishtar Hospital Multan, 30 minutes after he was allegedly shot to the abdomen by armed robbers. Examination revealed that the patient was in hypovolemic shock with the blood pressure of 90/60 mmHg, weak peripheral pulses at the rate of 140 per minute, cold peripheries, and Glasgow Coma Score (GCS) of 13/15. His chest was clear, but his abdomen was tense and diffusely guarded. On removing the pressure dressing done by the rescuing ambulance staff, an actively bleeding entry wound was observed on the left lumbar region roughly 5 cm lateral to the umbilicus and the exit wound on his back at L4 level 4 cm medial to anterior superior iliac spine. Despite immediate resuscitation with two liters of warm crystalloid, the patient remained hemodynamically unstable with minimal urine output. Within the next 20 minutes, the patient was wheeled to the operating room and explorative laparotomy was undertaken via midline incision.

Intraoperative findings included a 3x3 cm perforation of anterior wall of the third part of duodenum, multiple perforations in ascending colon, and a zone I retroperitoneal hematoma. Massive hemorrhage ensued when the plane of the hematoma was entered by Kocher maneuver and by medial mobilization of colon. The field immediately got flooded with blood. Hemostasis was obtained by direct gentle pressure by assistant's finger over IVC against the spine above and below the rent. Approximately 3,000 ml of blood was suctioned out to find a 5-cm longitudinal tear in the infrarenal part of IVC, which was repaired by direct suturing with prolene (polypropylene) 5/0. Then the duodenal perforation was repaired primarily by vicryl (polyglactin 910) 2/0, and retrocolic gastrojejunostomy was made. Perforated colon was removed, and double barrel colostomy was made. 

**Figure 1:**
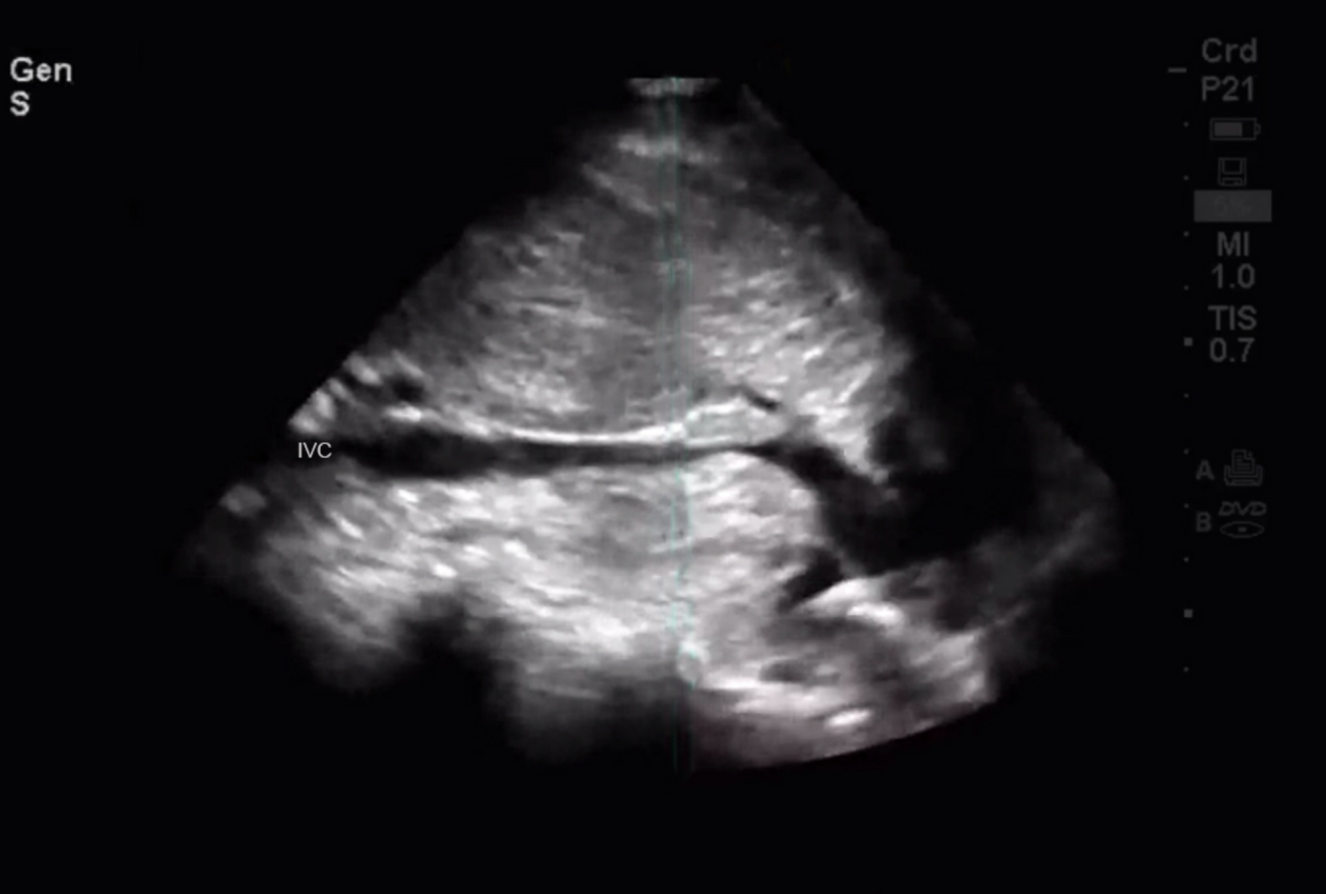
Ultrasonographic image of patient's patent inferior vena cava.

He was transfused 2,500 ml of packed cells and 1,000 ml of plasma intra- and postoperatively. For the first three postoperative days, he remained in the intensive care unit, after which he was shifted to the general ward. Ceftriaxone and metronidazole were given as antimicrobial cover and clexane (enoxaparin) 40 mg daily for the first four postoperative days for anticoagulation. No evidence of thrombosis or extension of the defect was seen on the ultrasound done on the sixth postoperative day (Figure 1).

His chief postoperative complaint was pain at the wound site, which was managed accordingly. The rest of his stay at the hospital remained uneventful and was discharged after 16 days of stay in the hospital. The patient remained stable on successive follow-ups, and colostomy was reversed successfully after 16 weeks.

## Discussion

The decrease in mortality rates in patients with abdominal IVC injuries has only been marginal over the past many decades. While 30% to 50% of cases with IVC injury exsanguinate to death before reaching the hospital, another 50% die despite the maximal possible hospital care [3]. Multiple factors predict the outcome of the patient in IVC injuries, including the mechanism of trauma, systolic blood pressure on presentation, response to resuscitation, part of IVC injured, and concomitant solid organ and vascular injuries [4,5]. Nonsurvivors reportedly have hemodynamic instability, a lower GCS, severe blood loss, increased transfusion requirements, and need for thoracotomies and other extensive operative procedures [6]. Anatomically, the infrarenal segment of IVC is the most frequently injured part and fortunately carries a better prognosis as compared to suprarenal or retrohepatic segments [4,6].

Our patient survived because of his early shifting to the hospital, control of intraabdominal bleed by tamponade effect of the hematoma, and the infrarenal part of IVC being involved. As proven in other studies, these good predictors resulted in the survival of our patient.

The primary intervention needed in IVC injuries is the control of hemorrhage as active bleed badly affects prognosis [7,8]. As in our case, retroperitoneal hematomas produce a tamponade effect on IVC, stopping further bleed, which increases the likelihood of survival. The study done by Millikan showed a 91% survival rate of patients with retroperitoneal tamponade against 93% mortality rate of patients without tamponade [4]. Multiple cases have been reported where exploration of retroperitoneum started torrential bleed by removal of the tamponade effect of hematomas and death of a patient on table [2]. A study done by Huerta and Nguyen showed that 40% of the patients died of uncontrollable bleeding after the tamponade effect was released [3]. Hence, exploration or no exploration remains debatable. Naraslmman and Okyere, in their studies, concluded that in the presence of a contained hematoma causing tamponade, a conservative approach might be opted instead of exploration [7,8].

Different techniques have been published in the literature for the repair of IVC, including primary repair by direct suturing i.e., venorrhaphy, ligation, grafting, portocaval shunting, atriocaval shunting, splenorenal anastomosis, and venacaval transposition [3,9-11]. Before choosing the procedure, merits and demerits should be considered for each in every patient subjectively. IVC repair with direct suturing may lead to the narrowing of the IVC and deep vein thrombosis [12,13]. Nevertheless, most authors recommend it even if the luminal diameter is compromised [14]. In a study done by Buckman, there was only a 5% incidence of pulmonary emboli and a 10% incidence of thrombosis in patients who underwent IVC repair by venorrhaphy [2]. On the contrary, ligation, especially when done in the suprarenal part, leads to multiple complications, including hemodynamic instability, decreased cardiac output, renal compromise, and massive pedal edema [13,14]. The infrarenal segment can be ligated with fewer complications and better survival [2]. The use of grafts has been discouraged because of the high occlusion and thrombosis rates [9]. Similarly, the use of shunts has been condemned as well, as they can be disastrous if done by inexperienced trauma team [9].

In our patient, grafting or shunting was not possible due to a lack of facilities, required equipment, and specialized vascular surgeons. Ligation could be done as a last resort, but the direct repair was preferred because the infrarenal part was accessible, and lumen diameter looked wide enough. This proved to be a better choice in concordance with other studies, which preferred direct repair to ligation. Our patient also had concomitant visceral injuries needing surgical intervention. This correlates with other studies that report that other viscera are invariably damaged by penetrating injuries reaching IVC and should be operated accordingly [2,3].

## Conclusions

Despite the standardization of resuscitation and operative approaches over the last many decades, IVC injuries carry very high mortality and morbidity, even in specialized trauma units. Delayed transport of patients to the hospital, delay in diagnosis, poor resuscitation, failure control hemorrhage, and inadequate damage control surgery result in dismal outcomes. Radical improvements made in these areas will hopefully improve the survival rates of patients with such extensive vascular injuries.

In countries like Pakistan, where hospitals are far and wide, have limited facilities, and are enormously overloaded, patients with such injuries rarely survive. There is a need to establish fully equipped trauma units with specialized surgical teams to cope up with such challenging cases. Since surgeons continue to encounter vascular and abdominal trauma, open and endovascular techniques will hopefully evolve constantly, giving us promising results in the coming future.
